# Prevalence and Predictors of Silent Myocardial Ischemia in Diabetic Patients

**DOI:** 10.7759/cureus.82407

**Published:** 2025-04-16

**Authors:** Hafiz Abdul Manan, Hammad Rauf Chishti, Franklin Dzigbodi Hewlett

**Affiliations:** 1 Cardiology, London Northwest University Healthcare NHS, London, GBR; 2 Cardiology, Punjab Institute of Cardiology, Lahore, PAK; 3 Internal Medicine, Jinnah Hospital Lahore, Lahore, PAK; 4 General Medicine, Kharkiv National Medical University, Kharkiv, UKR

**Keywords:** cardiovascular risk, diabetes mellitus, dyslipidemia, glycemic control, hypertension, non-invasive screening, silent myocardial ischemia

## Abstract

Background: Silent myocardial ischemia (SMI), a frequently underdiagnosed condition in diabetic patients, contributes significantly to increased cardiovascular morbidity and mortality. The absence of typical symptoms, such as angina (particularly in diabetic individuals with altered pain perception due to autonomic neuropathy), allows ischemic episodes to go unnoticed, while chronic hyperglycemia, endothelial dysfunction, oxidative stress, and low-grade inflammation further predispose them to SMI. Yet despite its serious consequences, it often remains undetected due to both symptomlessness and limitations in standard screening, highlighting the urgent need for proactive risk stratification and targeted diagnostic approaches.

Objective: To determine the prevalence and key risk factors associated with SMI in diabetic adults, aiming to enhance early detection through targeted risk stratification and inform tailored screening strategies to reduce cardiovascular events in this high-risk group.

Methodology: This cross-sectional study was conducted at Jinnah Hospital, Lahore, from May 2020 to May 2021. A total of 162 diabetic patients aged ≥40 years with a minimum five-year duration of diabetes and no prior history of overt coronary artery disease, ascertained through clinical history review and verification from available medical records, were enrolled using convenient sampling. This approach was selected due to time and resource constraints within the clinical setting. All participants underwent comprehensive evaluation, including detailed medical history, physical examination, laboratory tests (HbA1c and lipid profile), and cardiac assessments using myocardial perfusion imaging, exercise stress testing, and resting ECG. Multivariate logistic regression analysis was applied to identify independent predictors of SMI, with results reported as odds ratios (OR) and 95% confidence intervals (CIs). A p-value of <0.05 was considered statistically significant.

Results: SMI was detected in 61 (37.65%) diabetic patients. Multivariate logistic regression analysis identified several independent predictors of SMI: smoking (OR: 2.67; 95% CI: 1.32-5.41; p = 0.006), hypertension (OR: 1.89; 95% CI: 1.02-3.51; p = 0.041), dyslipidemia (OR: 2.15; 95% CI: 1.18-3.93; p = 0.012), diabetes duration >10 years (OR: 2.34; 95% CI: 1.22-4.48; p = 0.010), and poor glycemic control (HbA1c >7%) (OR: 3.21; 95% CI: 1.59-6.49; p = 0.001). Advancing age was also significantly associated with increased SMI risk (OR per year: 1.05; 95% CI: 1.02-1.09; p = 0.003), with a marked rise in prevalence observed beyond the age of 55. These findings highlight a high burden of silent myocardial ischemia among diabetic individuals and underscore the importance of identifying key predictors to support early detection, targeted screening, and risk-based management strategies to mitigate adverse cardiovascular outcomes.

Conclusion: SMI affects over one-third of diabetic individuals, often going undetected due to the absence of symptoms and limitations in routine screening. Key risk factors include smoking, poor glycemic control, long-standing diabetes, dyslipidemia, hypertension, and increasing age-particularly beyond 55 years. Incorporating non-invasive cardiac assessments such as stress ECG and myocardial perfusion imaging into screening protocols for high-risk diabetics can enable earlier diagnosis, timely intervention, and ultimately reduce the burden of future cardiovascular events.

## Introduction

A severe illness that is often misdiagnosed, silent myocardial ischemia (SMI) is defined by asymptomatic myocardial ischemia, which increases the risk of unfavorable cardiovascular events [1]. Since SMI does not exhibit the characteristic angina sign of conventional ischemic heart disease, it is difficult to diagnose and often delays prompt care [2]. Individuals with diabetes mellitus, a group known to have altered pain perception owing to autonomic dysfunction, have a much greater incidence of SMI [3]. Consequently, individuals with diabetes may experience ischemic episodes without chest discomfort, making them more vulnerable to heart failure, sudden cardiac events, and death [4]. Studies have shown that diabetics with SMI are two to four times more likely to develop major cardiovascular events compared to non-diabetics [4].

SMI remains underdiagnosed largely due to its asymptomatic nature and the limitations of routine cardiovascular screening in asymptomatic individuals. Standard evaluations like resting ECG or symptom-based assessments frequently fail to detect myocardial ischemia in diabetic patients without classic anginal symptoms. Moreover, inadequate utilization of more sensitive diagnostic modalities, such as stress testing or myocardial perfusion imaging, contributes further to the low detection rates in this high-risk group.

Diabetes mellitus, a major risk factor for cardiovascular disease (CVD), also causes endothelial dysfunction and exacerbates atherosclerosis and microvascular complications [5]. Chronic hyperglycemia in diabetes creates a pro-inflammatory and oxidative environment that accelerates vascular injury and impairs coronary microcirculation, thereby reducing myocardial perfusion even without obstructive coronary lesions [6]. These changes predispose the myocardium to ischemia that can occur silently. Additionally, diabetic autonomic neuropathy, a frequent complication of long-standing diabetes, blunts the afferent pain pathways responsible for ischemic pain signaling [7]. As a result, ischemic episodes may progress unnoticed, allowing SMI to develop undetected. These interconnected mechanisms explain why individuals with diabetes are particularly prone to asymptomatic ischemia and highlight the urgency of early detection strategies to prevent major cardiovascular outcomes such as myocardial infarction, arrhythmias, and heart failure [8].

Recognizing the frequency and drivers of SMI in this cohort is of paramount importance as diabetes is becoming a growing public health problem worldwide [9]. In numerous studies, diabetics have shown varying prevalence rates of SMI (ranging from 20% to 50%) due to heterogeneity in research populations, screening methods, and risk factor profiles [10]. Common determinants of SMI include chronic diabetes, sub-optimal glycemic control, the presence of other comorbidities such as hypertension and dyslipidemia, smoking, and microvascular/macrovascular complications. To develop region-specific risk predictors, further study is necessary since the proportional contributions of these variables vary across populations [11].

Identifying individuals most at risk for SMI remains a clinical challenge due to its asymptomatic nature and the limitations of conventional screening methods. Resting ECG, though commonly used, lacks the sensitivity needed to detect ischemia in asymptomatic diabetic patients. In contrast, stress testing and MPI provide greater diagnostic accuracy for uncovering subclinical myocardial ischemia. Given the elevated cardiovascular risk faced by diabetics with undetected SMI, understanding its prevalence and key risk factors is crucial. The objectives of this study were: (1) To estimate the prevalence of SMI in diabetic adults, including both type 1 and type 2 diabetes; and (2) To identify independent risk factors associated with SMI using statistical modeling. By addressing these objectives, the study aims to inform the development of targeted screening strategies and risk stratification models to support earlier diagnosis, timely intervention, and ultimately a reduction in adverse cardiovascular outcomes in this high-risk population.

Operational definitions

Type 1 diabetes, a chronic autoimmune condition characterized by the destruction of insulin-producing beta cells in the pancreas, leading to absolute insulin deficiency. It typically manifests in childhood or adolescence but can occur at any age.

Type 2 diabetes, a metabolic disorder resulting from insulin resistance and relative insulin deficiency, is often associated with obesity, a sedentary lifestyle, and genetic predisposition. It is the most common form of diabetes in adults.

Convenient sampling, a non-probabilistic sampling technique where participants are selected based on their accessibility and proximity to the researcher. This method is often used when time and resources are limited.

Stress test, a diagnostic procedure used to assess the heart's ability to respond to physical stress (e.g., exercise or pharmacological agents). It helps detect myocardial ischemia by monitoring ECG changes, heart rate, and blood pressure.

SMI, a condition characterized by objective evidence of myocardial ischemia in the absence of angina or other typical symptoms. It is often diagnosed using non-invasive diagnostic tools like stress tests or myocardial perfusion imaging.

MPI, a non-invasive imaging technique that uses radioactive tracers and gamma cameras to evaluate blood flow to the heart muscle. It is a sensitive method for detecting ischemia, especially in asymptomatic individuals.

Hypertension, a medical condition characterized by persistently elevated blood pressure, typically defined as systolic blood pressure ≥140 mmHg or diastolic blood pressure ≥90 mmHg.

Dyslipidemia, a condition marked by abnormal levels of lipids in the blood, including elevated LDL cholesterol, reduced HDL cholesterol, or elevated triglycerides, which increase the risk of cardiovascular disease.

HbA1c (glycated hemoglobin), a measure of average blood glucose levels over the past two to three months. Levels above 7% are typically considered indicative of poor glycemic control in diabetic patients.

## Materials and methods

Study design and setting

This cross-sectional study was conducted over a one-year period (May 2020 to May 2021) in the outpatient department of Jinnah Hospital, Lahore, which serves a large and diverse diabetic population drawn from both urban and peri-urban regions, making it a representative and practical setting for population-based research on diabetes-related cardiovascular risks. 

Inclusion and exclusion criteria

The study population consisted of adult diabetic patients aged 40 years or older with a confirmed history of either type 1 diabetes mellitus (T1DM) or type 2 diabetes mellitus (T2DM) for at least five years. Although T1DM and T2DM differ in their pathophysiological mechanisms, both conditions are associated with an elevated risk of CVD due to shared mechanisms such as endothelial dysfunction, chronic inflammation, autonomic neuropathy, and accelerated atherosclerosis, which also contribute to the development of SMI [12,13]. Inclusion of both diabetic subtypes allows for broader applicability of the findings and a more comprehensive understanding of SMI prevalence across the diabetic spectrum.

Participants were included only if they had no clinical evidence of symptomatic CAD. The absence of symptoms was determined through a combination of detailed clinical history and review of available medical records, confirming that there were no prior reports of exertional angina, chest pain, or any diagnosed ischemic heart disease. Individuals with a documented history of myocardial infarction, percutaneous coronary intervention (PCI), or coronary artery bypass grafting (CABG) were excluded [14].

Additional exclusion criteria comprised patients with advanced renal impairment, defined as an estimated glomerular filtration rate (eGFR) of less than 30 mL/min/1.73 m², given the altered hemodynamic responses and increased cardiac event risk in this group [15]. Moreover, individuals with severe systemic illnesses that significantly impact prognosis and limit participation were excluded. These included cases with advanced malignancy, end-stage liver disease, severe heart failure classified as New York Heart Association (NYHA) Class III or IV, or progressive neurological disorders likely to impair physical performance or cardiac assessment reliability [16,17].

Sample size

A total of 162 eligible participants were enrolled using a convenience sampling technique from patients attending regular outpatient visits. This non-probability method was selected due to time and logistical constraints inherent in real-world clinical research settings, particularly in resource-limited environments such as public tertiary care hospitals [18]. Given the cross-sectional nature of the study and the aim to explore the prevalence and risk factors of SMI in a high-risk diabetic population, convenience sampling allowed timely recruitment and ensured access to a representative pool drawn from the hospital’s diverse urban and peri-urban patient base. Moreover, similar sampling strategies have been employed in prior observational studies on cardiovascular risk among diabetic populations in low- and middle-income countries, where randomized sampling is often not feasible due to logistical and resource barriers [19-20]. While we acknowledge that convenience sampling may introduce selection bias and limit the generalizability of findings [21], the study population encompassed a broad spectrum of diabetic individuals, contributing to external relevance. To mitigate potential bias, rigorous inclusion and exclusion criteria were applied, and data were collected systematically to preserve internal validity [22]. This pragmatic approach balances feasibility with the need for meaningful clinical insights in high-risk diabetic populations.

Data collection

All eligible participants were systematically screened for SMI regardless of their individual risk factor profile. This universal screening approach ensured uniform evaluation across the entire cohort and minimized selection bias. Each participant underwent a comprehensive clinical evaluation, comprising a detailed medical history, physical examination, and laboratory investigations.

The history included demographic characteristics, diabetes type and duration, smoking status, medication use, and comorbidities. Lifestyle factors such as tobacco and alcohol use, as well as physical activity, were assessed through structured, self-reported questionnaires during face-to-face interviews. In cases of ambiguity or inconsistent reporting, information was cross-verified using available medical records. No biochemical validation (e.g., serum cotinine) was performed for smoking status.

Physical examination findings were complemented with laboratory assessments including HbA1c, lipid profiles, serum creatinine, and blood pressure measurements. Microvascular complications, retinopathy, nephropathy, and peripheral neuropathy, were evaluated based on fundoscopic findings, urine albumin-to-creatinine ratio, and clinical neuropathic symptoms, respectively.

To assess for SMI, all participants underwent a standardized, stepwise non-invasive cardiac evaluation. Initially, a resting 12-lead ECG was performed in the supine position to identify baseline ischemic changes, arrhythmias, or conduction abnormalities. This was followed by treadmill-based exercise stress testing using the Bruce protocol, during which participants were monitored for exercise-induced ST-segment changes, heart rate response, and clinical symptoms under physician supervision with emergency support available. For individuals with inconclusive or non-interpretable exercise stress results, or those physically unable to perform treadmill testing, MPI was carried out using a technetium-99m sestamibi single-photon emission computed tomography (SPECT) protocol. MPI involved either pharmacologic (adenosine or dipyridamole) or exercise-induced stress followed by radionuclide imaging to detect perfusion defects indicative of myocardial ischemia.

Statistical analysis

Data analysis was performed using SPSS software (version 25.0, IBM Corp., Armonk, NY). Continuous variables were first assessed for normality using the Shapiro-Wilk test. Normally distributed variables were expressed as mean ± standard deviation (SD), whereas non-normally distributed variables were presented as medians with interquartile ranges. Categorical variables were summarized using frequencies and percentages. The prevalence of SMI was determined by calculating its proportion within the study population.

To identify independent predictors of SMI, chi-square tests were used for categorical variables and independent-samples t-tests for continuous variables. Variables with p-values <0.1 in univariate analyses, along with clinically relevant covariates, were entered into a multivariate logistic regression model. The model was adjusted for potential confounders, including age, sex, duration of diabetes, hypertension, dyslipidemia, smoking status, glycemic control (HbA1c), and presence of microvascular complications. Results were reported as adjusted odds ratios (ORs) with corresponding 95% confidence intervals (CIs).

There was no missing data in the dataset, as all variables were fully recorded for each participant. Therefore, no imputation or data exclusion due to missing values was required. Model performance was evaluated using classification accuracy and receiver operating characteristic (ROC) curve analysis. The area under the curve (AUC) was used to assess the model’s discriminative power. A p-value of less than 0.05 was considered statistically significant.

Ethical approval

This study was approved by the Ethical Review Board of Jinnah Hospital Lahore (JHL/DM/ERB/238), and written informed consent was obtained from all participants. Participants were fully informed of potential discomforts or risks before testing. All procedures were conducted in accordance with the Declaration of Helsinki. Given that the study involved non-invasive cardiac evaluations, including exercise stress testing and MPI, particular attention was paid to minimizing potential risks. Patients were screened for contraindications to exercise testing, and all stress procedures were conducted under the supervision of trained medical personnel with appropriate resuscitation equipment available on-site. The risk-benefit ratio was carefully considered, especially for asymptomatic individuals, and the study protocol was designed to align with standard clinical practice for high-risk diabetic populations.

## Results

The research participants (n=162) had an average HbA1c level of 8.10 ± 1.30%, indicating poor glycemic control (Table 1). A tendency toward prehypertension was evident, with a mean systolic blood pressure of 138.60 ± 17.50 mmHg and a mean diastolic pressure of 82.40 ± 11.20 mmHg, both approaching the upper limits of the prehypertensive range. Notably, 38.27% of the cohort met the diagnostic criteria for hypertension based on systolic or diastolic thresholds. Lipid profile assessment revealed elevated cardiovascular risk: the mean total cholesterol was 210.30 ± 42.70 mg/dL, and LDL cholesterol averaged 128.70 ± 36.10 mg/dL, with half the participants exceeding 130 mg/dL. The average HDL cholesterol was 42.60 ± 10.90 mg/dL, with 42.59% of individuals falling below the protective threshold of 40 mg/dL. Furthermore, mean triglyceride levels were 187.20 ± 54.80 mg/dL, with 63.58% of participants exceeding the 150 mg/dL cutoff, confirming a substantial burden of dyslipidemia in the study population (Table 2).

**Table 1:** Baseline Characteristics of Study Participants (n=162) T1DM: type 1 diabetes mellitus; T2DM: type 2 diabetes mellitus; CVD: cardiovascular disease

Characteristic		Number of Patients (n; %)	Mean ± SD
Age (years)		-	58.40 ± 9.70
Gender	Male	92 (56.79%)	-
	Female	70 (43.21%)	-
Type of diabetes	T2DM	145 (89.51%)	-
	T1DM	17 (10.49%)	-
Duration of Diabetes (years)		-	11.20 ± 5.30
HbA1c (%)		-	8.10 ± 1.30
Comorbidities and Lifestyle Factors	Hypertension	104 (64.20%)	-
	Dyslipidemia	87 (53.70%)	-
	Smoking	49 (30.25%)	-
	Obesity (BMI ≥ 30)	61 (37.65%)	-
	Family history of CVD	43 (26.54%)	

**Table 2:** Laboratory Investigations and Clinical Parameters SBP, systolic blood pressure; DBP, diastolic blood pressure; TC, total cholesterol; LDL, low-density lipoprotein cholesterol; HDL, high-density lipoprotein cholesterol; TG, triglycerides.

Parameter	Mean ± SD	% Above Clinical Cutoff	Reference Range (Standard)
HbA1c (%)	8.10 ± 1.30	74.69% (≥7%)	Normal: <5.7%; Diabetes: ≥6.5%
SBP (mmHg)	138.60 ± 17.50	38.27% (≥140 mmHg)	Normal: <120; Hypertension: ≥140
DBP (mmHg)	82.40 ± 11.20	34.57% (≥90 mmHg)	Normal: <80; Hypertension: ≥90
TC (mg/dL)	210.30 ± 42.70	56.79% (≥200 mg/dL)	Desirable: <200; High: ≥240
LDL cholesterol (mg/dL)	128.70 ± 36.10	50.00% (≥130 mg/dL)	Optimal: <100; High: ≥160
HDL cholesterol (mg/dL)	42.60 ± 10.90	42.59% (<40 mg/dL)	Low (Risk): <40; Protective: ≥60
TG (mg/dL)	187.20 ± 54.80	63.58% (≥150 mg/dL)	Normal: <150; High: ≥200

**Figure 1:**
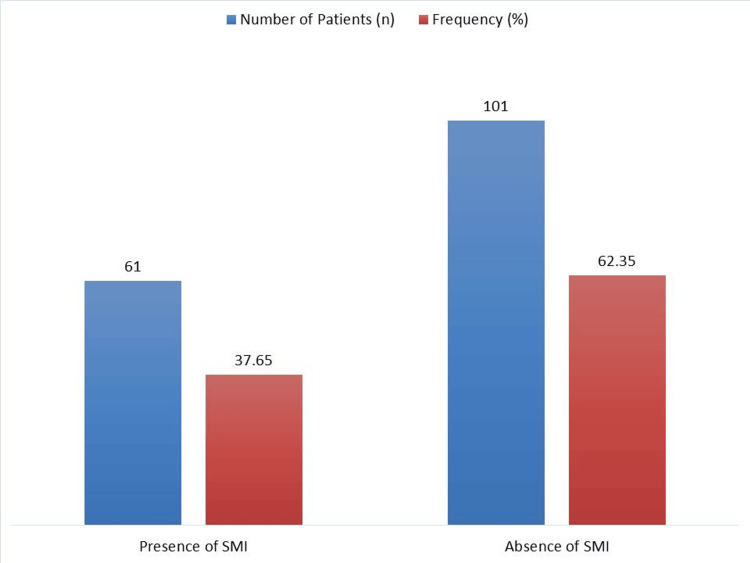
Prevalence of Silent Myocardial Ischemia (SMI) in Diabetic Patients

Among the 162 research participants, 37.65% (61 patients) had SMI, while 62.35% (101 patients) did not (Figure 1).

**Figure 2:**
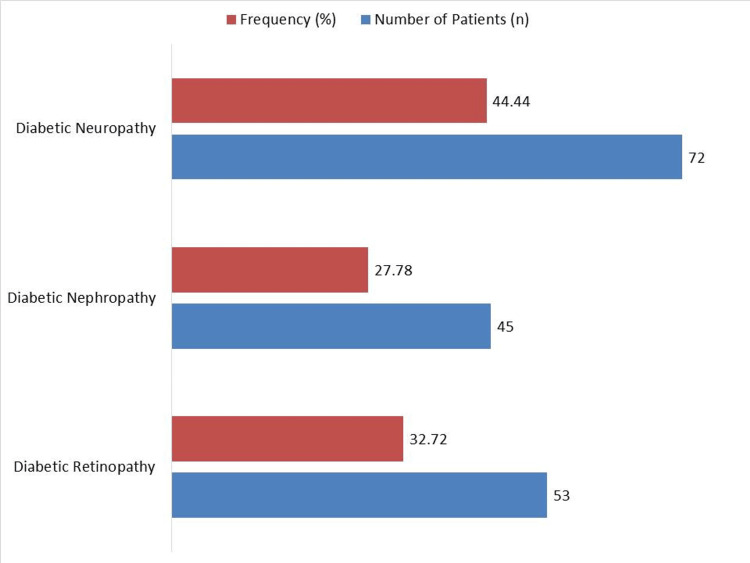
Presence of Microvascular Complications in Study Population

There is a significant burden of microvascular problems among diabetes patients, as shown by the 72 (44.44%) research participants who had diabetic neuropathy, 53 (32.72%) who had diabetic retinopathy, and 45 (27.78%) who had diabetic nephropathy (Figure 2).

The following factors were shown to be significant independent predictors of SMI in diabetic patients: smoking (p=0.006), age (p=0.003), diabetes duration over 10 years (p=0.010), hypertension (p=0.041), dyslipidemia (p=0.012), and poor glycemic control (HbA1c >7%, p=0.001), shown in Table 3. Among them, smoking (OR: 2.67, 95% CI: 1.32-5.41), long-term diabetes (OR: 2.34, 95% CI: 1.22-4.48), and HbA1c >7% showed the highest correlation with SMI (OR: 3.21, 95% CI: 1.59-6.49). These results emphasize how cardiovascular risk factors and inadequate metabolic management contribute to the development of SMI in diabetics.

**Table 3:** Associated Factors of Silent Myocardial Ischemia (Multivariate Logistic Regression Analysis)

Predictor	Odds Ratio (95% CI)	p-value
Age (per year increase)	1.05 (1.02–1.09)	0.003
Duration of Diabetes (>10 years)	2.34 (1.22–4.48)	0.010
Hypertension	1.89 (1.02–3.51)	0.041
Dyslipidemia	2.15 (1.18–3.93)	0.012
Smoking	2.67 (1.32–5.41)	0.006
HbA1c > 7%	3.21 (1.59–6.49)	0.001

**Figure 3:**
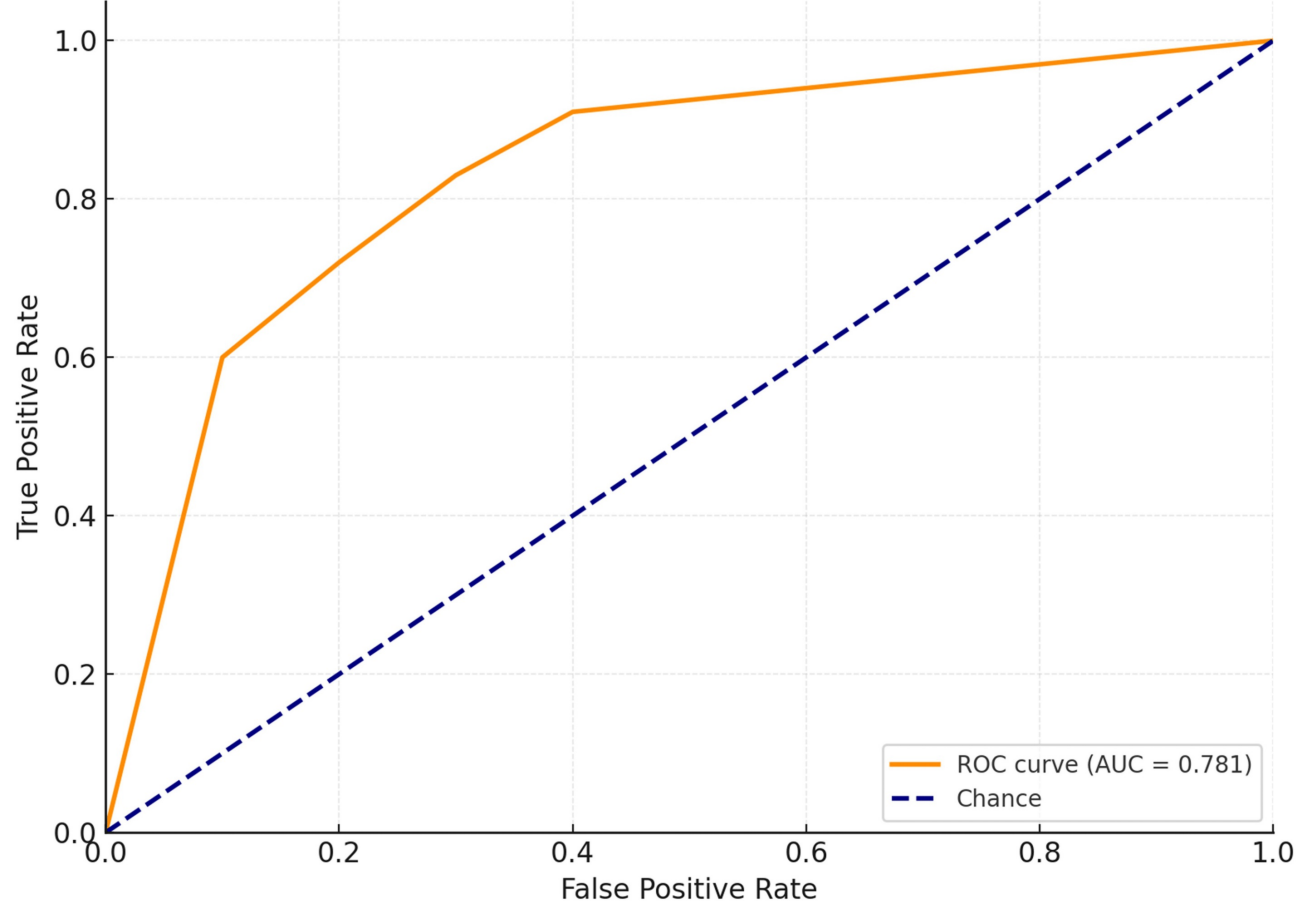
Receiver Operating Characteristic (ROC) Curve Analysis

To evaluate the discriminative ability of the logistic regression model in predicting SMI, a ROC curve analysis was conducted (Figure 3). The full model, incorporating all significant predictors, yielded an AUC of 0.781 (95% CI: 0.715-0.848), with a sensitivity of 76.1% and specificity of 70.3%, indicating good overall model performance. An AUC of 0.781 indicated a 78.1% probability that the model would correctly differentiate between a diabetic patient with SMI and one without, thereby reinforcing the clinical utility of the model beyond p-values and odds ratios.

## Discussion

The current study revealed that 37.65% of asymptomatic individuals with T2DR had SMI, highlighting a significant hidden cardiovascular burden. This prevalence is consistent with previous literature, where SMI rates among diabetic populations range from 20% to 40% [23,24]. However, the observed prevalence in our study is on the higher end of this spectrum. This difference can be attributed to several factors, primarily differences in methodology and population characteristics. Nomura et al. reported a prevalence of approximately 20% using adenosine-stress MPI, which is a more sensitive but less accessible technique [24]. Additionally, their cohort may have been enriched for individuals with a higher pretest probability of SMI. In contrast, our study employed exercise stress ECG, a less sensitive but more accessible and widely used screening tool, and was applied to a broader, high-risk diabetic population. These differences in diagnostic modality, patient selection criteria, and overall risk burden may explain the higher prevalence observed in our cohort.

Among the evaluated risk factors, poor glycemic control, as indicated by an HbA1c greater than 7% [25], was the strongest predictor of SMI (OR: 3.21, 95% CI: 1.59-6.49, p = 0.001), consistent with prior studies linking chronic hyperglycemia to endothelial dysfunction, oxidative stress, and accelerated atherosclerosis [26]. The role of glycemic variability, which refers to the fluctuations in glucose levels, is increasingly recognized as an independent contributor to vascular injury. It is possible that these fluctuations exacerbate silent ischemic episodes beyond the effects of sustained hyperglycemia alone. The elevated mean HbA1c of 8.10% in our cohort underscores the need for improved glycemic control in order to mitigate cardiovascular risk in T2DM patients. This aligns with previous research emphasizing the importance of both glycemic control and variability in managing long-term diabetic complications [27].

Duration of diabetes was also significantly associated with SMI (OR: 2.34, 95% CI: 1.22-4.48, p = 0.010), supporting the notion that prolonged exposure to hyperglycemia increases the burden of atherosclerosis and other vascular complications. Long-standing diabetes likely accelerates both macrovascular and microvascular damage, further predisposing individuals to asymptomatic ischemia, as also stated in previous studies [1,28,29]. This finding highlights the importance of early and sustained management of diabetes to prevent cardiovascular morbidity.

Hypertension and dyslipidemia are significant independent predictors of SMI in individuals with T2DM. Our study found a typical atherogenic lipid profile, characterized by low HDL-C, high LDL-C, elevated total cholesterol, and triglycerides, which is consistent with diabetic dyslipidemia. This dyslipidemia is known to contribute to the increased cardiovascular risk in T2DM, with disturbances in fatty acid metabolism leading to an abnormal lipoprotein cascade, from large chylomicrons to small, dense LDL and HDL particles. Solano et al. [30] highlight the need for comprehensive management, emphasizing lifestyle changes alongside statin therapy to reduce cardiovascular risk. Similarly, Tomkin [31] suggests that achieving normal glycemia can help reverse fatty acid metabolism abnormalities, but this is difficult to maintain as the disease progresses. Tomkin further discusses the potential role of newer lipid-lowering drugs, such as ezetimibe and MTP inhibitors, in treating diabetic dyslipidemia. While statins remain the cornerstone of treatment, these novel agents may offer additional benefits, particularly in individuals with persistent dyslipidemia despite statin use. Tomkin’s focus on dyslipidemia's complex lipid metabolism in diabetes complements our findings, emphasizing the importance of early and aggressive management to reduce cardiovascular events in diabetic patients. In our cohort, the combination of lipid-lowering agents and comprehensive interventions, including diet, exercise, and glycemic control, is essential for improving lipid profiles and reducing the cardiovascular burden associated with T2DM.

Smoking was another significant predictor of SMI (OR: 2.67, 95% CI: 1.32-5.41, p = 0.006), consistent with the Klein et al [32] well-established link between smoking and endothelial dysfunction, as well as increased inflammatory markers [32]. While 30.25% of participants reported smoking, quantitative data, such as pack-years, were not captured, which could have provided more detailed insights into the dose-dependent relationship between smoking and SMI. This limitation suggests that future studies may benefit from including more precise data on smoking intensity.

Given the high prevalence of SMI and the identified risk factors, we recommend that screening for SMI in asymptomatic diabetic patients be considered, particularly using cost-effective and accessible tools like exercise stress ECG in resource-limited settings. While current guidelines from organizations such as the American Diabetes Association (ADA) [25] and the European Society of Cardiology (ESC) [33] do not universally recommend routine SMI screening, our findings suggest that targeted screening in high-risk individuals could be beneficial. These results point to a potential gap in current clinical practice, indicating the need for updated recommendations that incorporate risk stratification to guide cardiovascular screening strategies in diabetes.

Strengths and limitations

In terms of study strengths, one of the key advantages of this research is its focus on a clinically underrecognized yet high-risk condition, SMI, within a diabetic population. By utilizing a multimodal, non-invasive cardiac evaluation strategy, which included stress testing, resting ECG, and myocardial perfusion imaging, the study enhances diagnostic accuracy and provides practical insights into the real-world application of these tools. The inclusion of both Type 1 and Type 2 diabetic patients, while considering their differing pathophysiological profiles, ensures that the study's findings are broadly applicable to diverse diabetic populations.

However, several limitations warrant attention. First, the cross-sectional design of the study limits our ability to make causal inferences regarding the relationship between risk factors and SMI. Second, the convenience sampling from a single tertiary care center introduces potential selection bias, which may limit the generalizability of the findings to broader populations. Despite this, we took steps to mitigate selection bias by employing strict inclusion and exclusion criteria. Third, while lifestyle factors such as smoking were assessed through structured self-reports, they were not biochemically validated, introducing the potential for reporting bias. Lastly, although we controlled for major clinical confounders, the possibility of residual confounding cannot be fully ruled out.

Future research should focus on multicenter, longitudinal studies that include stratified diabetic cohorts and utilize probability-based sampling to improve the generalizability of the results. Additionally, integrating more invasive diagnostic modalities could help confirm and expand upon the findings, providing more definitive evidence on the relationship between diabetes and SMI.

## Conclusions

This study identifies several critical factors contributing to the concerning prevalence of SMI in diabetic patients, including poor glycemic control, long-standing diabetes, hypertension (HTN), dyslipidemia, smoking, and older age. These findings underscore the importance of targeted cardiovascular screening, especially for high-risk diabetic patients-those with multiple comorbidities, poor glycemic control, or a long duration of diabetes.

We recommend repeated cardiovascular screening at regular intervals (e.g., annually or biennially) for these high-risk individuals, as early detection of SMI through non-invasive diagnostic methods (such as stress ECG and myocardial perfusion imaging) can lead to timely interventions and significantly reduce the risk of unfavorable cardiac events. Since SMI is often asymptomatic, early screening can help mitigate long-term cardiovascular morbidity and mortality in this population.

For older adults with diabetes, addressing modifiable cardiovascular risk factors is crucial to reducing their overall risk. Better management of blood pressure, cholesterol levels, and glycemic control, along with smoking cessation, can substantially lower the likelihood of cardiovascular events. By improving these factors, older diabetic patients can significantly decrease their cardiovascular risk and improve long-term health outcomes.
